# Coexistence of Sarcoidosis and Systemic Sclerosis

**DOI:** 10.1155/2013/684216

**Published:** 2013-12-05

**Authors:** Senol Kobak, Fidan Sever, Oya Sivrikoz, Ahmet Karaarslan

**Affiliations:** ^1^Department of Rheumatology, Faculty of Medicine, Sifa University, Bornova, 35100 Izmir, Turkey; ^2^Department of Pulmonary Diseases, Faculty of Medicine, Sifa University, 35100 Izmir, Turkey; ^3^Department of Pathology, Faculty of Medicine, Sifa University, 35100 Izmir, Turkey; ^4^Department of Ortopedics, Faculty of Medicine, Sifa University, 35100 Izmir, Turkey

## Abstract

Sarcoidosis is a multisystem granulomatous disease characterized by hilar lymphadenopathy, involvement of internal organs, and diverse skin lesions. Systemic sclerosis is an autoimmune disease characterized by skin hardening and different internal organ fibrosis, including vascular abnormality. Immune response associated with Th-2 has been shown in the early and active stage of the disease. In this paper, we report coexistence of systemic sclerosis with sarcoidosis in a female patient presenting with granulomatous dermatitis, interstitial lung disease, and Raynaud's phenomenon complaints.

## 1. Introduction

Sarcoidosis is a systemic disease characterized by the involvement of multiple tissues and organs and a noncaseating granulomatous reaction, which is not well understood [1]. Although its pathogenesis is not clear, there appears to be a cellular immune system activation and a nonspecific inflammatory response against some genetic and environmental factors [2]. Th1 and macrophages caused by proinflammatory cytokines induce the inflammatory cascade; the formations of granulomas occur as a result of tissue permeability, cellular influx, and local cell proliferation [3]. The key pathological finding of sarcoidosis are noncaseating epitheloid cellular granulomas [4]. Epitheloid cells are transformed bone marrow monocytes with significant secretory activity. Increased active CD4 T lymphocytes have been shown in tissues with sarcoidosis. These lymphocytes visibly increase in the granulomatous lesion, while small numbers of CD8 T-Ly, B-cells, plasma, and mast cells are identified on the outer part of the granuloma [5]. A difference in prevalence, clinical findings, and the course of disease in varied races and ethnic groups suggests that sarcoidosis is a heterogeneous disease [6]. The disease is more prevalent in women and tends to develop after 40 years of age. The incidence of sarcoidosis in the USA is 10.9 per 100.000 in the white race and increases to 35.5 per 100.000 in African-Americans, with a more severe course [7]. Sarcoidosis is a chronic granulomatous disease that may display different clinical findings. The disease most frequently presents with bilateral hilar lymphadenopathy, infiltrations in the lungs and skin, and eye lesions. It may mimic a number of primary rheumatic diseases and/or develop associated to these [8]. The incidence of sarcoidosis together with different connective tissue diseases (RA, SLE, and Sjögren's syndrome) has increased. Similar immunological abnormalities observed between sarcoidosis and autoimmune diseases suggest a possible common and similar etiopathogenesis. Coexistence of sarcoidosis with systemic sclerosis (SS) has been reported in a few patients. In this paper, a rare case of sarcoidosis and SS coexistence is reported.

## 2. Case Report

**Figure 1:**
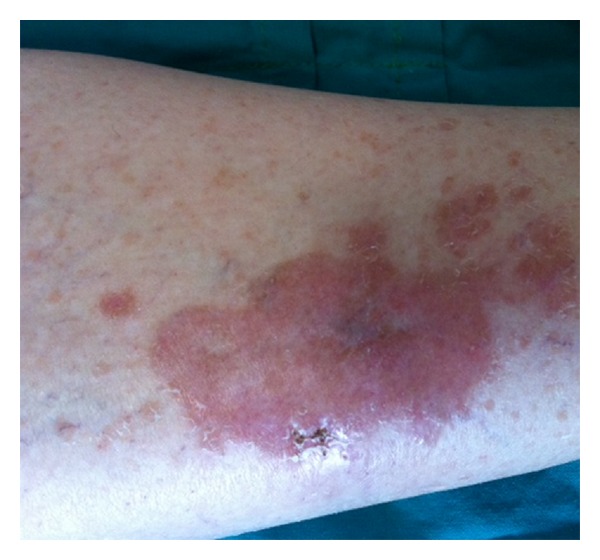
Brown-red skin lesion on the right foot pretibial site not protruding from the skin.

We present 52-year-old woman, who did not seek medical care for complaints that continued for six years and suggested Raynaud's phenomenon in the finger joints of the hands. One year ago, brown-red skin lesions developed at the pretibial part of the right foot and the patient consulted a dermatologist. However, the patient did not receive any results from the dermatological examination and visited our rheumatology polyclinic after arthralgia, morning stiffness, and effort dyspnoea started together with the Raynaud's complaints. Upon a physical examination, telangiectasia on the face, lessening of the mouth opening, sclerodactilia and Raynaud's phenomenon pallor phase of the fingers, and a brown-red skin lesion of approximately 15 cm on the right foot pretibial site not protruding from the skin were found Figure 1. In the auscultation of the lungs, crepitant rales were determined on both lungs. On laboratory examinations, the erythrocyte sedimentation rate was determined to be 38 mm/h (normal <20 mm/h) and the C-reactive protein levels to be 3.5 mg/dL (normal 0–0.5 mg/dL), and the rheumatoid factor (RF) was negative. Liver and kidney function tests were normal. A routine urinalysis was performed and was found to be normal. On serological examination, the antinuclear antibody (ANA): 1/320 nucleolar and homogenous pattern anti-Scl70 antibody were positive. The complement components C3 and C4 were normal, whereas anti-CCP, anti-Ro, Anti-La, anti-Sm, and anti-ribosomal P antibodies were determined to be negative. Serum angiotensin converting enzyme (ACE) level was 65 U/L (normal: 8–52 U/L), serum calcium level was 10.2 mg/dL (normal: <9.8 mg/dL), and the serum hidroxy-D3 was normal. On the lung graphy, a thin reticular pattern was observed. On the thorax HRCT, ground glass opacification in line with interstitial lung disease and honeycomb appearance was observed. As a result, a thoracic diseases specialist was consulted. A bronchoscopy and BAL were performed and mixed (neutrophilic and lymphocytic) alveolitis was found. A skin biopsy was taken and the noncaseating granulomatous structure of the skin was consistent with granulomatous dermatitis, sarcoidal type. No acid-fast bacilli in the Ziehl-Neelsen histochemistry and fungus in the PAS histochemistry were observed. Structure consistent with leishmania was not determined in the Giemsa histochemistry. Tissue samples examined with PCR did not exhibit acid resistant bacilli (ARB). Based on the clinical, laboratory, and histological outcomes, the patient was diagnosed with sarcoidosis and SS. Corticosteroid 16 mg/day, Hydroxychloroquine 200 mg/day, and Azathioprine 150 mg/day were initialized. During the follow-up visits, the effort dyspnoea of the patient decreased and a significant improvement in the functional lung study (spirometry and DLCO) and in the skin lesions was observed. At six months, a decrease in active alveolitis lesions was observed on the control thorax HRCT and the chronic lesions were reported to remain stable. Regular patient follow-up continues.

## 3. Discussion

Sarcoidosis is a systemic, Th1-mediated granulomatous disease, with unknown etiology, involving more than one organ. Sarcoidosis may mimic a number of rheumatic diseases or may coexist side by side. It can commonly present with connective tissue diseases, including primary Sjögren's syndrome, SLE, and RA, including clinical findings that are indicative of vasculitis and/or spondyloarthritis. Enzenauer and West have shown that 6 out of 569 patients followed with connective tissue disease developed concomitant sarcoidosis within 10 years [9]. Although rarely seen, coexistence of sarcoidosis with SS was recorded in the literature. De Bandt et al. reported in 5 patients coexistence of sarcoidosis with SS [10]. They described that patients who have both sarcoidosis and SS have a poorer prognosis, but they were not able to make a generalization due to the small number of patients. Hasson and Singh-Grewal reported sarcoidosis coexisting with SS in a 3-year-old girl and mentioned that the case was the first case of a child to be recorded in the literature [11]. Sakamoto et al. reported coexistence of sarcoidosis with SS and PBS and suggested that there might be a common and similar cellular immunity defect in all three diseases [12]. Similarly, Suga et al. reported in a female case coexistence of sarcoidosis with SS, determined by skin and muscle biopsy [13]. It is essential to support the diagnosis of sarcoidosis with biopsy, together with the presence of hilar lymphadenopathy, systemic, and/or respiratory symptoms. Therefore, other pathologies and diseases causing granulomatous disease should also be excluded. In our case, diagnosis of sarcoidosis was done based on skin lesions, high serum ACE, and calcium levels and typical noncaseating granulomas determined in the skin biopsy. Other diseases, including tuberculosis, causing granulomatous dermatitis were prevented. Diagnosis of SS was made based on the presence of typical findings (Raynaud's phenomenon, sclerodactilia, telangiectasia, lung involvement, and anti-Scl-70 positivity). Skin involvement can be seen in both diseases. SS may cause skin hardening, together with typical skin lesions (morphea, etc.). Skin lesions observed in sarcoidosis include lupus pernio and granulomatous dermatitis. As in our case, a differential diagnosis should be made with skin biopsy. Interstitial lung disease is a common diagnosis in both diseases and clinical, radiological, laboratory, and bronchoscopic differentiation may be difficult [14]. The thorax computed tomography can reveal hilar lymphadenopathy presence; the high rates of CD4/CD8 in the bronchoscopy and in the BAL fluid may lead to a conclusion in favor of sarcoidosis. But this differentiation is important in terms of prognosis and therapy. Whereas SS with lung involvement may be progressive and fatal, lung involvement associated with sarcoidosis is more benign. For lung involvement resulting from sarcoidosis, only monitoring is recommended due to spontaneous remissions particularly in stages 1 and 2. In more advanced involvements, use of corticosteroids (CS) and immunosuppressive (IS) drugs are considered. However, in the presence of active alveolitis associated with SS, CS together with IS like Cyclophosphamide and Azathioprine are administered. The disease may in some cases be progressive and fatal, despite the therapy mentioned above. In this case, a low dose of CS (scleroderma renal crisis was taken into consideration) and Azathioprine was initialized. As a result of this treatment, a considerable regression was observed in the lung and skin findings.

Coexistence SS with sarcoidosis is an entity that should be rarely seen, when the incidence of both diseases are considered (50–400/million/year and 20/million/year, resp.) [15]. Moreover, the Th1/Th2 paradigm was present. Sarcoidosis is a Th1-mediated disease, whereas Th2 is predominantly present in the early and active stage of SS [16]. The underlying cause for this coexistence is theoretically expected to be rare, although it could be a common genetic, environmental, and pathogenetic mechanisms unknown at present. According to some researchers, this is not a true sarcoidosis, and thus it is sometimes known as a sarcoidosis-like disease. To support this claim, these researchers give the example that following silica exposure, hilar and mediastinal LAP develops in patients with SS. Granulomatous formations developing in association with silica should be considered in the differential diagnosis of sarcoidosis.

As a result, we are reporting the coexistence of sarcoidosis with SS. The overlapping of SS with sarcoidosis is a very rare entity. The Th1/Th2 paradigm is one of its most important causes. Since they both can exhibit similar clinical manifestations, it is a crucial difference to the overlapping syndromes for prognosis and therapy. Although the causes for this coexistence are not clear, further research is warranted to determine whether there is a common etiopathogenesis and/or coincidental coexistence.
